# Supplementary material for: Epitope-associated and specificity-focused features of EV71-neutralizing antibody repertoires from plasmablasts of infected children
Source: Nat Commun. 2017 Oct 2;8:762. doi: 10.1038/s41467-017-00736-9 (PMC5624920; doi:10.1038/s41467-017-00736-9)
Supplement: Supplementary file 1 — Supplementary Information [file 41467_2017_736_MOESM1_ESM.pdf]

Description of Supplementary Files

File Name: Supplementary Information
Description: Supplementary Figures, Supplementary Tables, Supplementary Methods and Supplementary References

File Name: Peer Review File

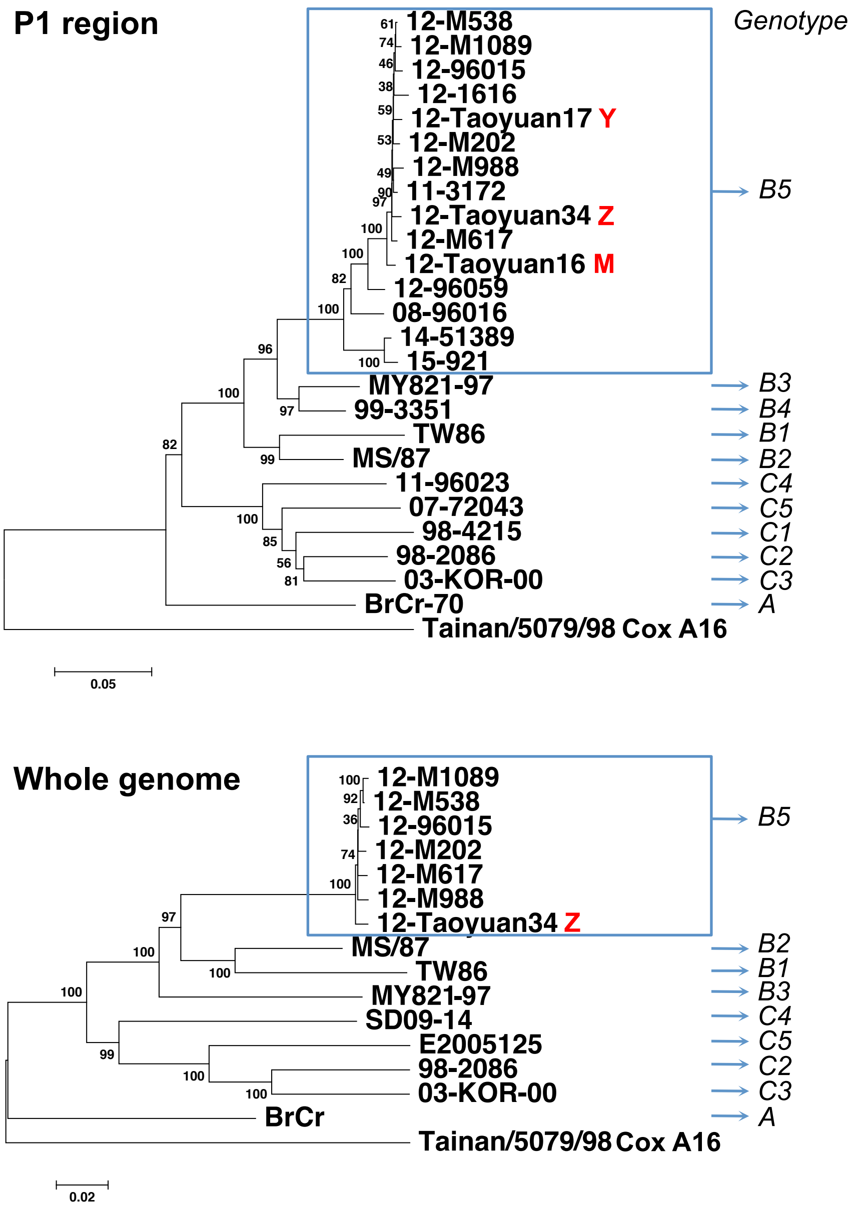

Supplementary Figure 1. Phylogenetic relationship of EV71 strains. Alignments of the complete P1 nucleotide sequences (2586 bp) and whole genome (7403 bp) were performed, and the phylogenetic trees were constructed using the neighbor-joining method and Kimura distance matrix model with 1,000 bootstrap replications in the MEGA program version 5.0. The P1 region encodes four structural proteins, VP4, VP2, VP3, and VP1. The P1 nucleotide sequences of the 2011 and 2012 genotype B5 isolates are at least 94.1% identical to one another, and the deduced amino acid

sequences are at least 98.3% identical to one another. The clinical isolates from donors M (12-Taoyuan16), Y (12-Taoyuan17), and Z (12-Taoyuan34) are highly related to each other (P1 nucleotide identity 98.4-99.1% and P1 amino acid identity 99.1-100%) and phylogenetically clustered into the B5 genotype of EV71. We also analyzed the complete genome obtained from the clinical isolate 12-Taoyuan34 from donor Z and other reference isolates collected in 2012. The 12-Taoyuan34 strain is at least 97.7% identical for the complete genome nucleotide sequence and at least 99.5% identical for the deduced amino acid sequence to all other 2012 EV71 reference isolates. These 2012 EV71 reference isolates, including 12-M538, 12-M1089, 12-M202, 12-M988, and 12-M617, have been published in a previous paper (1), and no obvious viral recombination between the 2012 EV71 isolates and other enteroviruses was detected. The sequences of 12-M538 (KF974790.1), 12-M1089 (KF974797.1), 12-96015 (KX267854.1), 12-M202 (KF974794.1), 12-M988 (KF974792.1), 11-3172 (KF154354.2), 12-M617 (KF974791.1), 08-96016 (GQ231942.1), MY821-97 (DQ341367.1), TW86 (FJ357380.1), MS/87 (U22522.1), 11-96023 (KX267855.1), SD09-14 (JX678883.1), E2005125 (EF063152.1), 98-4215 (JN874553.1), 03-KOR-00 (DQ341356.1), BrCr-70 (JN874547.1), and BrCr (U22521.1) EV71 viruses and the Tainan/5079/98 coxsackievirus A16 were retrieved from GenBank (1).

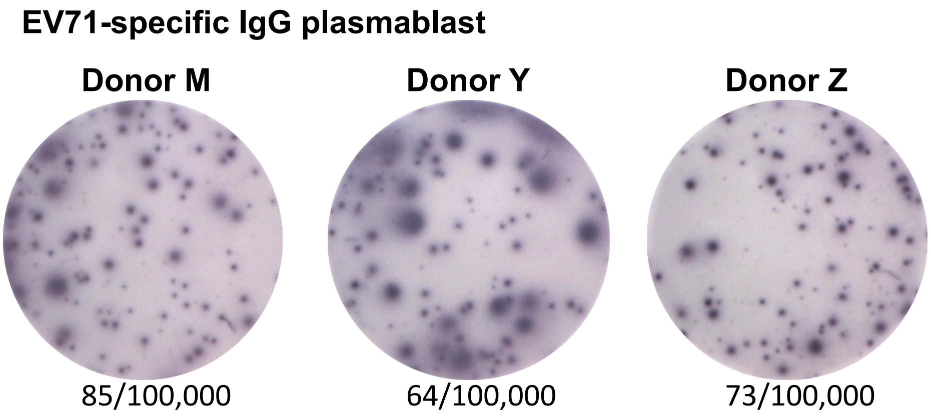

Supplementary Figure 2. EV71-specific IgG plasmablast responses to natural infection in three donors. The EV71-specific IgG plasmablasts were detected with an enzyme-linked immunosorbent spot assay. The ratio of EV71-specific IgG-producing cells to incubated peripheral blood mononuclear cells per well is shown in the figure. Each sample was tested in duplicate.

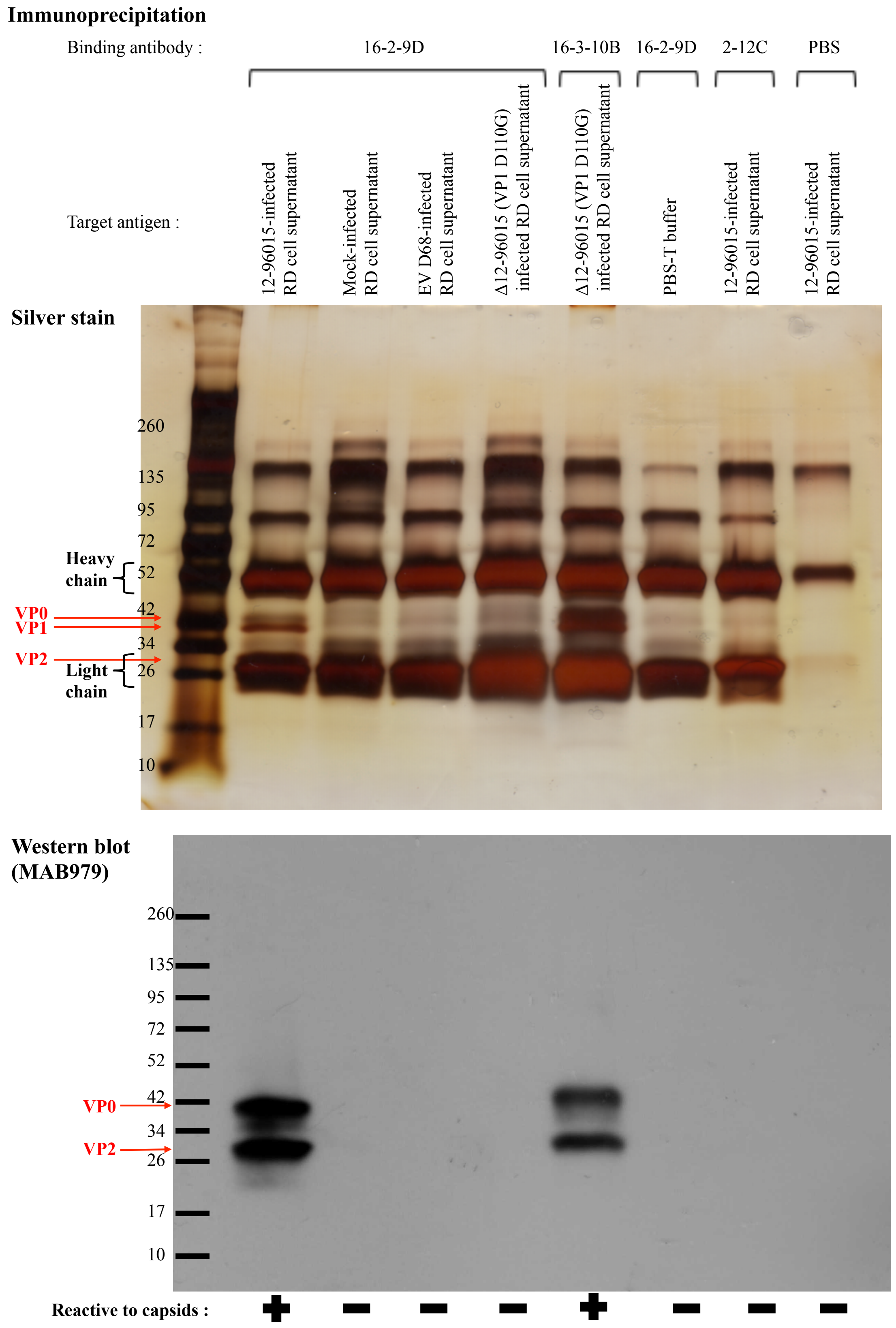

Supplementary Figure 3. EV71-specific mAbs immunoprecipitate viral capsids.

EV71-containing supernatants were prepared as antigens and reacted with bead-bound

antibodies. Eluates were separated by SDS-PAGE and examined by silver staining and western blot assay. The EV71 capsid protein VP0 (~42 kDa) and VP1 bands (~40 kDa) were visualized on a silver-stained gel. The stained human IgG heavy chains (~50 kDa) and light chains (~25 kDa) were detected on the gel. The VP2 and VP3 protein bands were probably mixed with IgG light chain bands. In the western blot analysis, EV71 capsids were detected by the anti-VP0/VP2 antibody MAB979 (1:1000 dilution, EMD Millipore). The figure shows that the EV71-specific antibodies 16-2-9D and 16-3-10B immunoprecipitated the 12-96015 viral capsid. Antibody 16-2-9D failed to react with its escape mutant harboring a single amino acid substitution (VP1 D110G). Instead, the binding of antibody 16-3-10B was unaffected by this escape mutation. The influenza monoclonal antibody 2-12C was used as a negative control antibody, and mock-infected and enterovirus D68-infected cell supernatants were used as antigen controls.

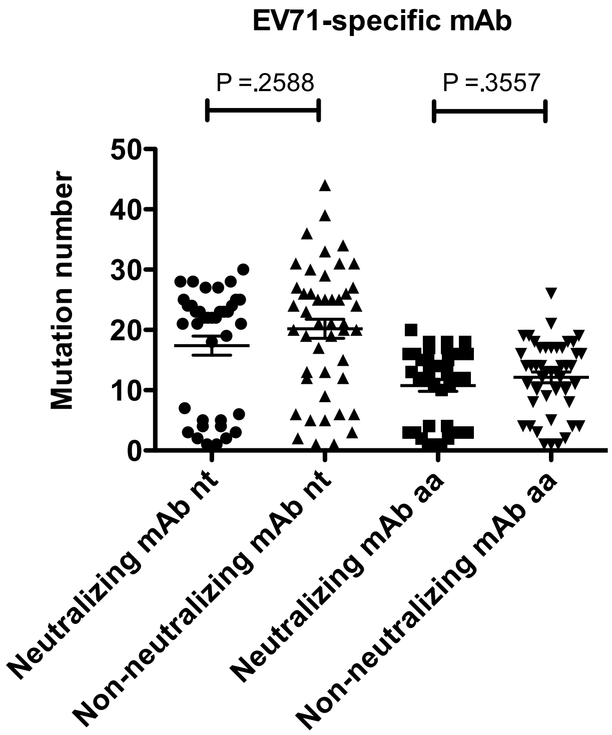

Supplementary Figure 4. Comparison of mutation numbers in the heavy chain variable domain between neutralizing and non-neutralizing EV71-specific antibody clones. Data are presented as the mean ± standard error. The difference in the number of mutations in the variable domains between two groups was examined by the Mann-Whitney test.

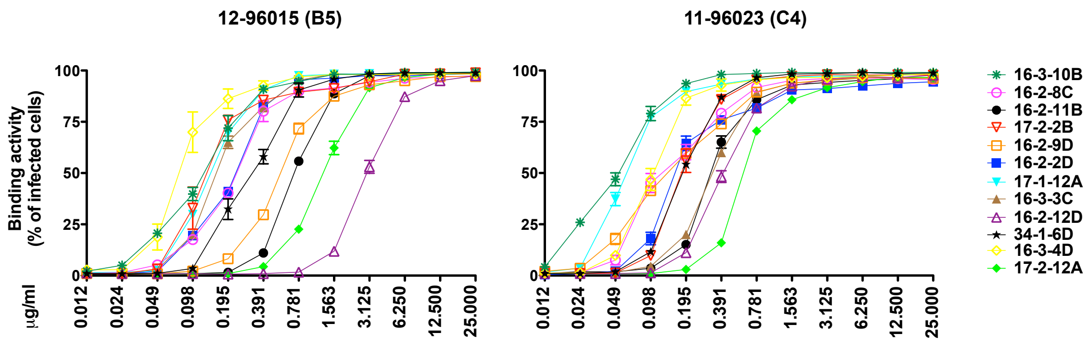

Supplementary Figure 5. Binding activities of neutralizing monoclonal antibodies to 12-96015 and 11-96023 EV71, as measured by flow cytometry. Data are presented as the mean ± standard error of the mean and represent two independent experiments.

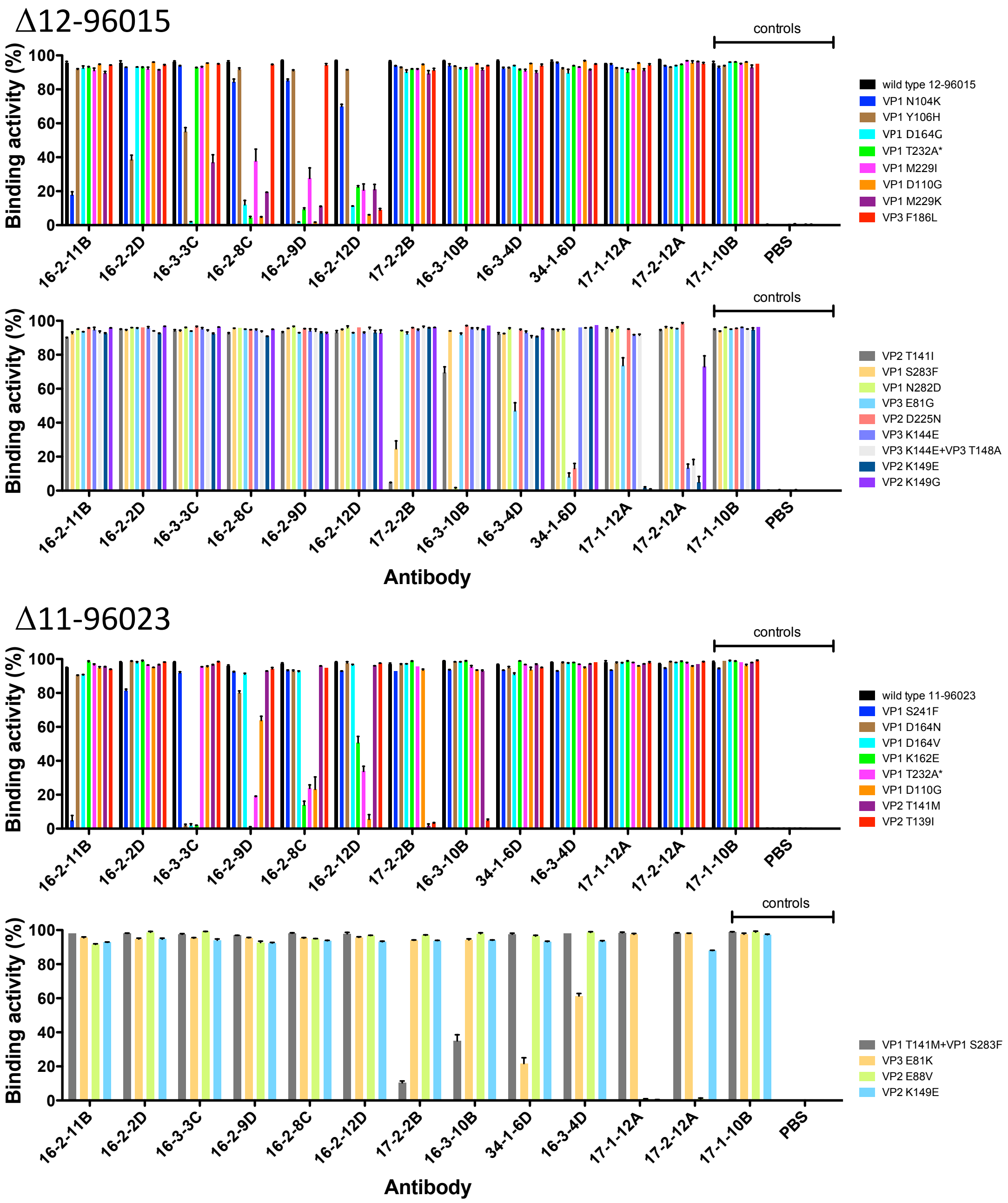

Supplementary Figure 6. Binding activities of EV71-neutralizing monoclonal antibodies to escape mutants. The non-neutralizing EV71-specific antibody 17-1-10B was unaffected by any of selected mutant viruses. Data are presented as the mean ± standard error of the mean and represent two independent experiments.

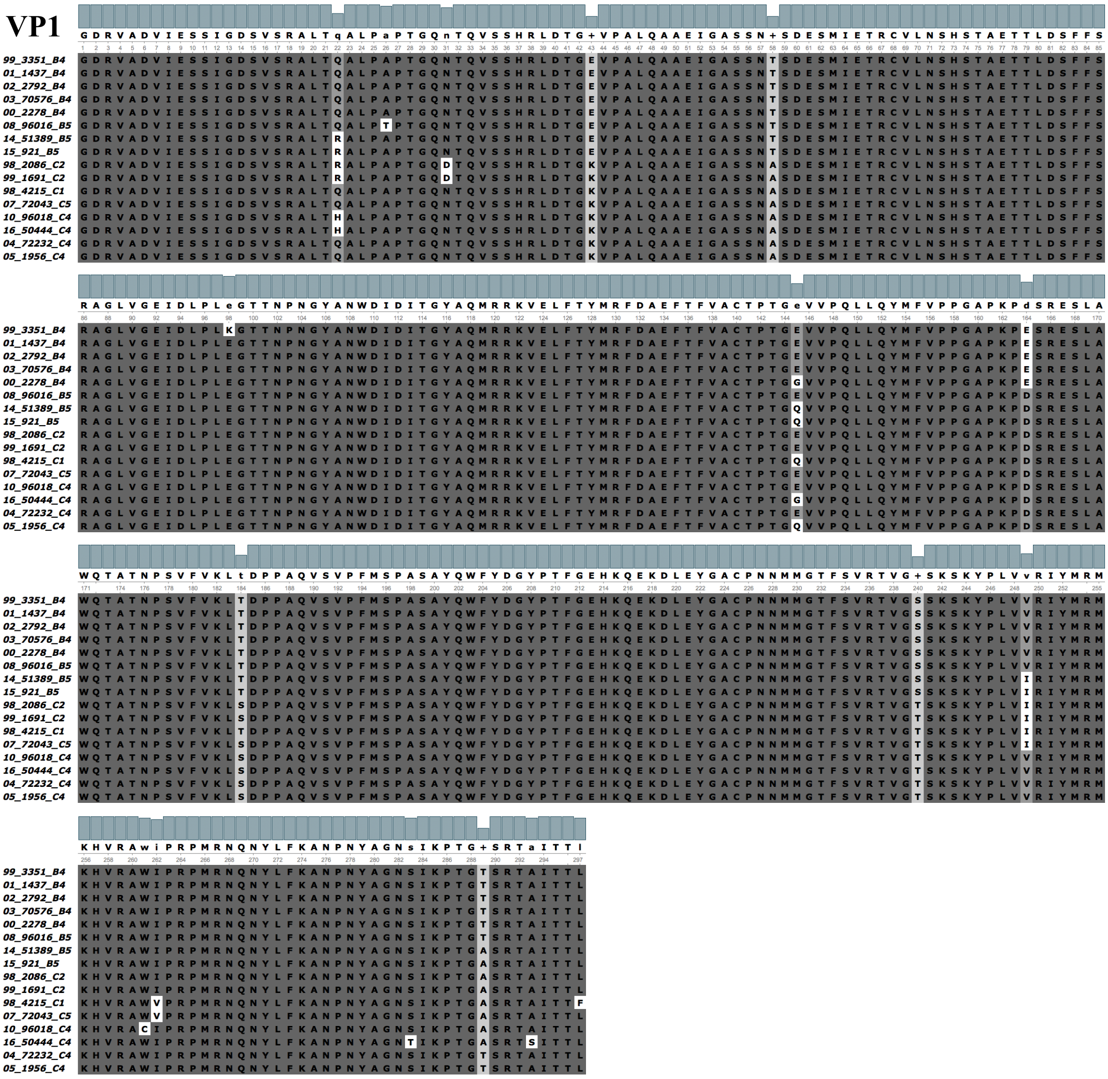
VP1

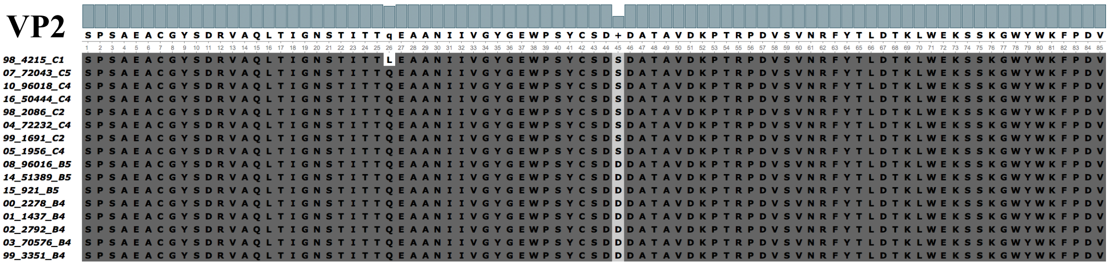
VP2

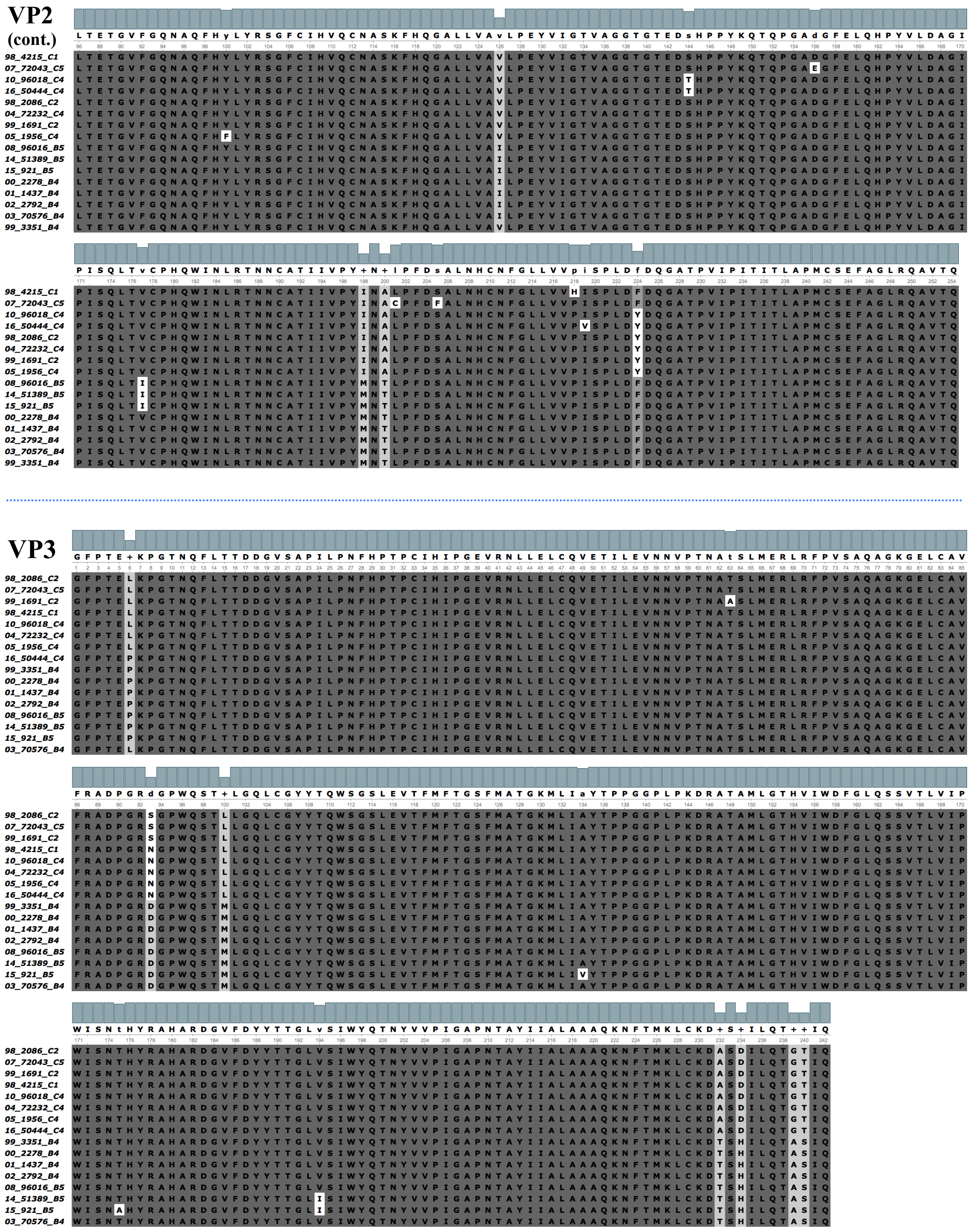

Supplementary Figure 7. Sequence alignment of the VP1, VP2, and VP3 proteins of the EV71 clinical strains from 1998-2016. Within the isolates, the percentage of amino acid differences are 6% for VP1, 6% for VP2, and 5% for VP3.

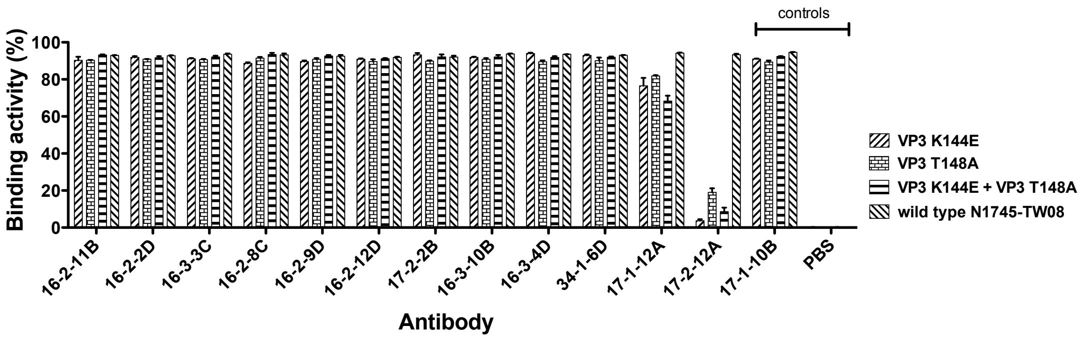

Supplementary Figure 8. Binding activities of neutralizing monoclonal antibodies to mutated EV71 N1745-TW08. One escape mutant selected from 12-96015 (genotype B5) with antibody 17-2-12A harbored two amino acid substitutions at VP3 residues 144 and 148. Antibody 17-2-12A selected another mutant with a single substitution at VP3 K144E. To determine the role of the VP3 T148A substitution in antibody binding and neutralization, a selection of site-specific mutations were introduced into the infectious cDNA clone for strain N1745-TW08 (genotype B5 EV71) (2). The VP3 K144E and VP3 T148A substitutions both individually and together abolished neutralization and binding by antibody 17-2-12A. The non-neutralizing EV71-specific antibody 17-1-10B was included in the test as a control. Data are presented as the mean ± standard error of the mean and represent two independent experiments.

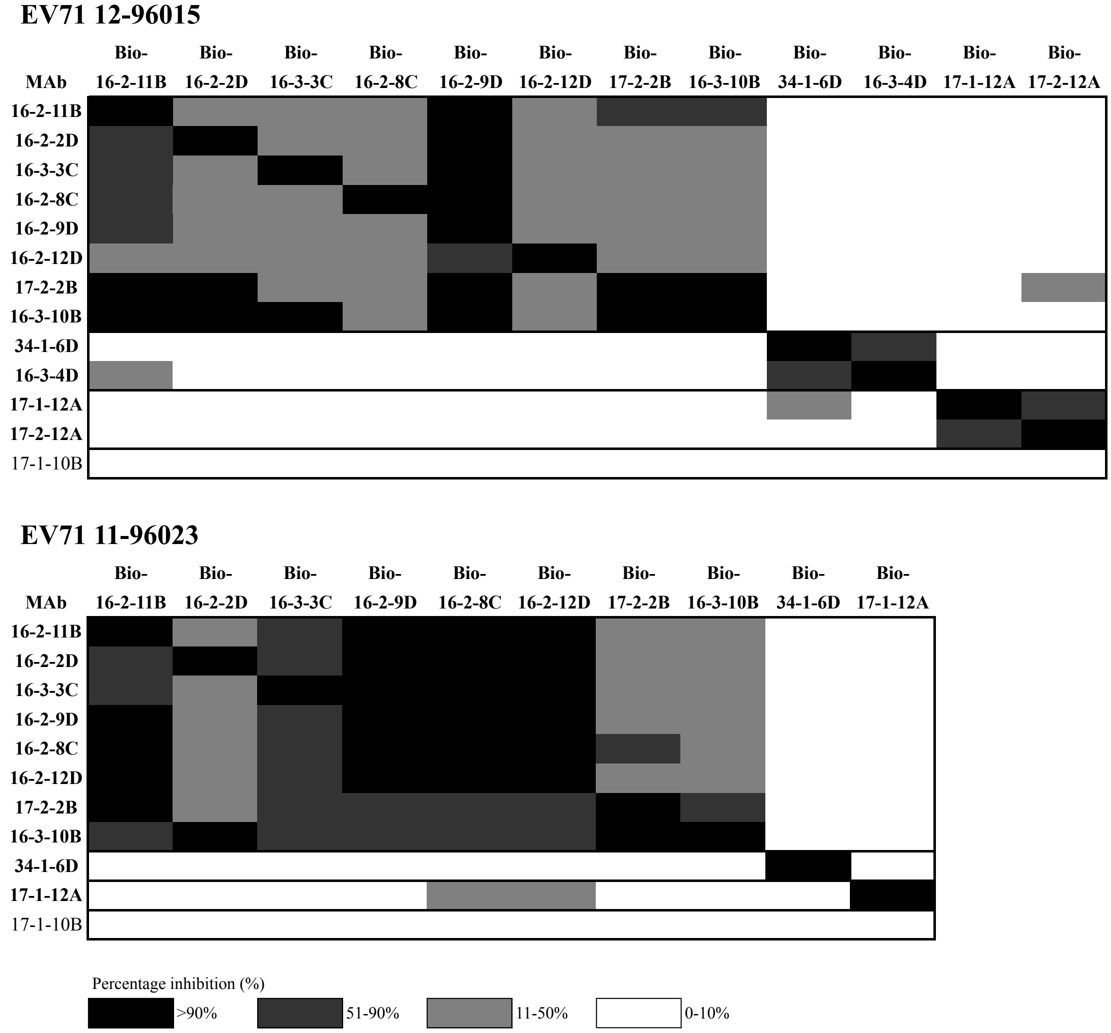

Supplementary Figure 9. Competitive binding to 12-96015 and 11-96023 EV71 by neutralizing monoclonal antibodies. A competition ELISA was used to determine the similarity in the binding sites between 12 representative neutralizing antibodies. A 10-fold molar excess of unlabeled antibody was used to compete against a biotinylated antibody for the binding to purified 12-96015 and 11-96023 viruses. The level of competition of each antibody in the binding assay against all other neutralizing antibodies is shown. All tests were performed in duplicate. The results show that canyon-specific antibodies competed with each other for binding, as did antibodies that targeted the 3-fold plateau and 2-fold plateau epitopes. The EV71-specific antibody 17-1-10B (non-neutralizing) did not compete for binding with any

member of this panel of neutralizing antibodies.

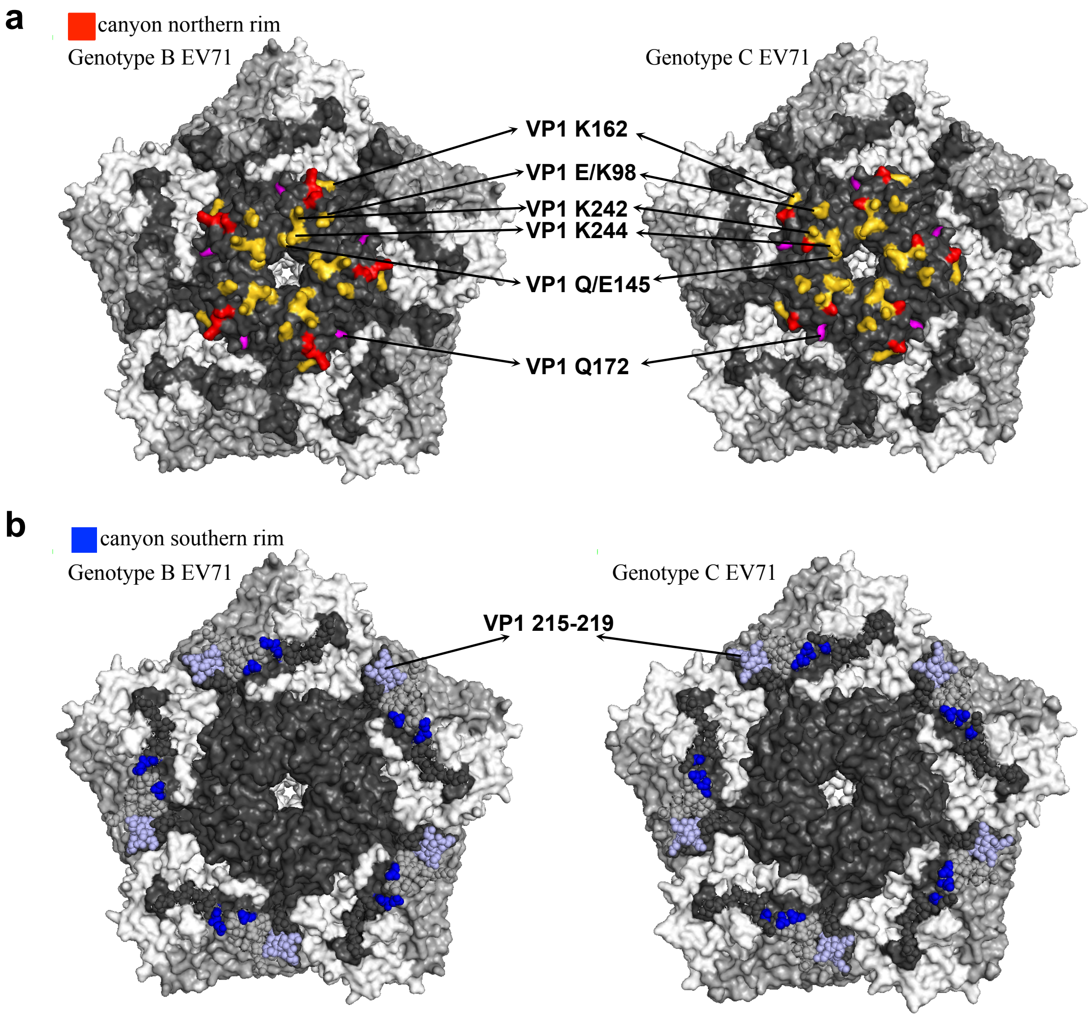

Supplementary Figure 10. EV71 capsid residues that are involved in binding to heparan sulfate, P-selectin glycoprotein ligand-1 (PSGL-1), and human scavenger receptor class B member 2 (hSCARB2). (a) The canyon northern rim epitope (colored in red) and proposed cell receptor binding sites were mapped onto the viral capsid based on the EV71 structures 3ZFF and 3VBS (3, 4). Previously reported residues that interacted with heparan sulfate and PSGL-1 (5, 6) are colored in gold, and the residue VP1 Q172 that interacted with hSCARB2 (7) is colored in magenta. **(b)** The canyon southern rim epitope (colored in blue) and proposed hSCARB2 binding sites (8, 9) were mapped onto the viral capsid based on the EV71 crystal structures 3ZFF and 3VBS (3, 4). The VP1 residues 215 to 219 at the canyon

southern rim are recognized by mouse monoclonal antibodies 22A12 (10, 11) and colored in light blue. The proposed hSCARB2 binding sites are located at VP2 residues 136 to 150 and VP1 residues 280 to 293 and are shown as spheres. All surface views of pentamers shown with the 5-fold vertex at the center were created using PyMOL, and VP1 is colored in black, VP2 is colored in grey, and VP3 is colored in white.

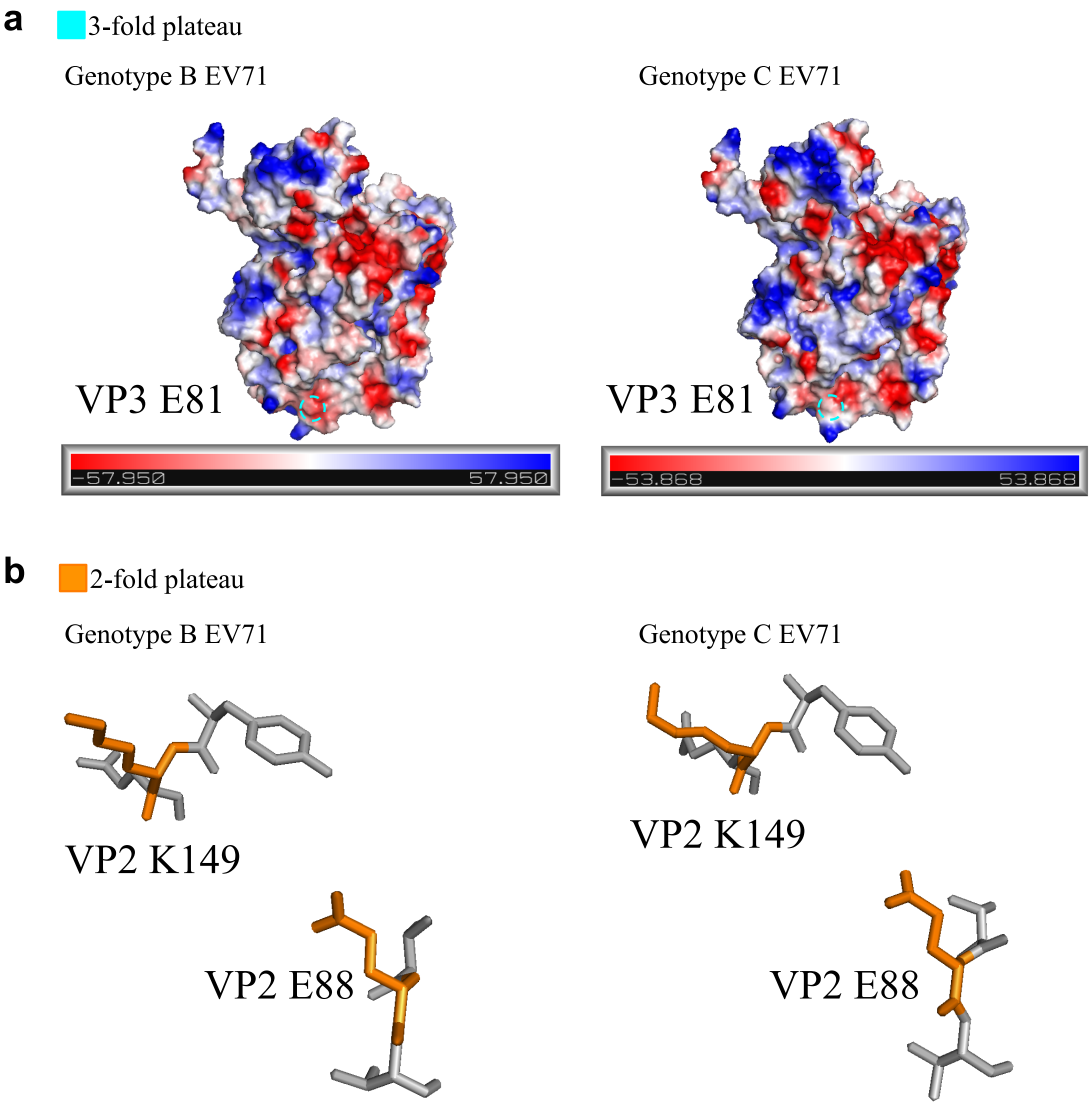

Supplementary Figure 11. Analysis of VP3 residue 81 of the 3-fold plateau epitope and VP2 residues 88 and 149 of the 2-fold plateau epitope. (a) The electrostatic potential at VP3 residue 81 of the 3-fold plateau epitope is more neutral in genotype C than that in genotype B EV71 based on previously reported structures. The escape mutant analysis showed that antibody 16-3-4D was sensitive to a single substitution of a negatively charged glutamate to a neutral glycine and had reduced binding to a mutation of a negatively charged glutamate to a positively charged lysine at the epitope. The preference of 16-3-4D for negatively charged residues at the 3-fold plateau epitope partially explains its loss of neutralization against genotype C4 viruses

in the study. The electrostatic potential surface map was created based on the EV71 structures 3ZFF and 3VBS (3, 4) using PyMOL. VP3 residue 81 is indicated by the cyan circle. **(b)** VP2 residue 149 of the 2-fold plateau epitope is the key amino acid for neutralization and binding for antibody 17-2-12A. The pattern of electrostatic potential is similar at the 2-fold plateau epitope between genotypes B and C EV71. However, the spatial orientation of the side chains of VP2 residue 149 is different between viruses, being more perpendicularly exposed in genotype B than in genotype C EV71. The amino acid structures at positions 88 and 149 of the VP2 protein were modeled based on the EV71 structures 3ZFF and 3VBS (3, 4) using PyMOL. The residues are shown as sticks, and residues 88 and 149 are colored in gold.

Supplementary Table 1. Non-neutralizing EV71 capsid-specific human monoclonal antibodies.

mAb	V_H	D_H	J_H	rf	Heavy chain Junction sequence	Mut#	λ/κ	$V_κ/V_λ$	$J_κ/J_λ$	Light chain Junction sequence	Mut#
Donor M											
16-3-12A	1-46*01 or 03	3-22*01	6*02	2	CARGPGPGGRYYYDSSDAYYYYGMDVW	25 (13)	λ	1-44*01	1*01	CATWDDSLNGYVF	9 (7)
16-3-2D	3-21*01	1-26*01	4*01 or 02 or 03	1	CARERVATTTGYFDIW	30 (14)	λ	3-21*03	3*02	CQVWDSRSDYRVF	17 (9)
16-2-2A	3-33*01 or 06	3-10*01	6*02	2	CARVNGRTIYGSGSYYHPEYYYGMDVW	25 (11)	κ	2-28*01 or 2D-28*01	4*01	CMETLQSRAVTF	6 (5)
16-2-8D	4-34*01 or 12	6-19*01	4*02	1	CATKATTSAWTAPDYW	21 (12)	λ	4-60*03	3*02	CETWDSNSRVF	12 (9)
16-3-7D	4-34*13	2-2*02	4*02	2	CATEATTSTWTAPDSW	25 (16)	λ	4-60*03	3*02	CETWDSNSRVF	24 (12)
16-2-1A	4-39*01	6-19*01	4*02	2	CARQPITAIAGIGGYW	26 (13)	λ	4-60*03	3*02	CETWDSNSWVF	15 (9)
16-2-12B	4-39*01	2-21*01	6*02	2	CARHKARSGFGYYYYGLDVW	39 (19)	λ	1-51*01	7*01	CGTWDSILSAAVF	19 (7)
16-2-7D	4-39*01	2-21*01	6*02	2	CARHKSRSGFGYYYYGLDVW	34 (19)	λ	1-51*01	7*01	CGTWDSILSAAVF	18 (7)
16-2-9B	4-39*01	6-19*01	6*02	2	CARHVPVAGFGYYYYGMDVW	21 (14)	λ	1-44*01	3*02	CAAWDDSLNNWVF	12 (9)
16-2-7A	4-39*01	6-25*01	6*02	1	CATHIPANGFGYYYYAMDVW	31 (17)	λ	3-21*03	3*02	CQVWERSSEHWVF	22 (12)
16-1-9A	4-39*01 or 02	4-17*01	6*02	3	CARHGTTMSTFGYYYYGMDVW	24 (17)	λ	1-47*01	3*02	CAAWDGSLTAWVF	13 (9)
16-3-12B	4-39*01 or 07	5-12*01	6*02	3	CARHLPRGVSPFGYYYYPMDVW	36 (18)	λ	1-51*01	1*01	CGAWDTRLSVYVF	23 (14)
16-1-1A	4-59*01	6-19*01	4*02	1	CARVAASRGGYPRPHFDLW	26 (14)	λ	2-14*01	2*01 or 3*01	CSSYTTTSVVF	12 (10)
16-3-6C	4-59*08	1-26*01	6*02	3	CARVLERRGPYYYYAVDAW	20 (14)	κ	3-15*01	3*01	CQQYNDWPTF	9 (4)
Donor Y											
17-1-10B	1-2*02	6-19*01	4*02	2	CARPVSGAMAFGYW	44 (26)	κ	4-1*01	4*01	CQQYFDIPLTF	21 (11)
17-3-4D	1-58*01	1-26*01	3*02	3	CASLAGGSYHDDFDIW	33 (21)	λ	3-27*01	3*02	CYSAADLNWGVF	16 (4)
17-2-12B	3-13*04	2-15*01	4*02	2	CARGFIDGRGGLDYW	24 (16)	λ	1-40*01	3*02	CQSYDRRLSGWVF	13 (8)
17-3-1B	3-15*01	5-18*01	6*02	2	CTGRIQRAVYYGMDVW	1 (1)	λ	1-36*01	3*02	CSTWDYSLSARVF	7 (5)
17-3-11B	3-21*01	2-15*01	4*02	3	CAPVGIVANW	3 (2)	λ	4-69*01	2*01 or 3*01	CQTWGTGIGVF	2 (0)
17-3-12A	3-23*04	1-14*01	3*01	1	CTKDLIWGTTEHERWSVLKGIINDASDVW	19 (17)	κ	1-17*01	1*01	CLQYSSYPRTF	25 (14)
17-3-4A	3-23*04	3-16*01	4*02	3	CGKSEETFGDILPHALDYW	17 (13)	λ	4-69*01 or 02	2*01 or 3*01	CQTWGTGIHDFF	13 (8)
17-3-2C	3-23*04	3-22*01	4*02	2	CARLHYSESSAYGSACDYW	13 (9)	λ	4-60*03	3*02	CETWDSHGRVF	13 (8)
17-2-6A	3-30-3*01	3-10*01	4*02	2	CARDADQFGSGTDYTDYW	31 (19)	λ	4-69*01	2*01 or 3*01	CQTWGSGVHVVF	26 (9)
17-2-2A	3-48*02	6-13*01	3*01 or 02	1	CARARGYSSSWYDAFDLW	31 (14)	λ	1-51*01	3*02	CEAWDSNLSGGVF	15 (10)
17-3-9C	3-48*03	3-22*01	4*02	2	CAPVDSSGYDSDYW	6 (3)	λ	4-60*03	3*02	CETWDINTWVF	10 (7)
17-3-5B	4-39*01	3-3*01	4*02	2	CASHLYYDFWSDYYNVDYW	20 (12)	λ	1-44*01	3*02	CAAWDDSLNGWVF	14 (11)
17-3-7B	4-39*01	3-3*01	4*02	2	CASHLYYDFWSDYYNVDYW	23 (14)	λ	1-44*01	3*02	CAAWDDSLNGWVF	12 (9)
17-3-7A	4-59*01	3-10*01	4*02	1	CASGQYYFDYW	6 (4)	λ	4-69*01	3*02	CQTWGTGIRVF	3 (1)
17-1-11B	4-61*02	6-19*01	4*02	1	CARGPSAYSSGWPFDSW	12 (8)	λ	1-40*01 or 02	3*02	CQSYDNSLSGWVF	8 (7)
17-3-8B	4-61*02	6-19*01	4*02	1	CARGPSAYSSGWPFDSW	15 (10)	λ	1-40*01 or 02	3*02	CQSYDISLSGWVF	10 (7)
17-2-8D	5-10-1*03	6-6*01	4*02	1	CARSSSSWTFGFDSW	26 (17)	κ	3-20*01	3*01	CQQYYDSPFTF	11 (8)
17-3-11C	5-10-1*03	6-6*01	4*02	1	CARSSSSWTFGFDYW	21 (14)	κ	3-20*01	3*01	CQQYYYSPFTF	6 (4)
Donor Z											
34-3-3C	3-7*01	4*02	3-10*01	1	CARLYFRAADYW	19 (8)	κ	2-30*02	1*01	CMQGTHWPWTF	5 (3)
34-3-4D	3-23*03	4*02	2-2*01	2	CARDGASRGPSRYPDYW	27 (16)	λ	4-69*02	2*01 or 3*01	CQTWGTGFQVF	14 (7)
34-3-11D	3-23*03	4*02	2-2*01	2	CARDGDSRGPSRYPDYW	25 (17)	λ	4-69*02	2*01 or 3*01	CQTWGTGFQVF	14 (12)
34-3-10A	3-23*03	4*02	3-22*01	2	CARDGDSRGPSRYPDYW	27 (18)	λ	4-69*02	2*01 or 3*01	CQTWTTGFQVF	16 (10)
34-3-7C	3-23*03	4*02	3-22*01	2	CARDGDSRGPSRYPDYW	29 (18)	λ	4-69*02	2*01 or 3*01	CQTWGTGFQVF	21 (14)
34-3-4C	3-23*04	4*02	6-19*01	1	CLNIGYSSGWNPGYW	9 (4)	λ	4-60*03	2*01 or 3*01 or 3*02	CETWDTNIRVF	3 (3)
34-3-8C	3-23*04	4*02	6-13*01	2	CAKILEAGGSDYW	6 (5)	λ	4-60*03	3*02	CETWDSNTRMF	1 (1)
34-3-10C	3-23*04	4*02	6-13*01	2	CAKILEAGGSDYW	13 (11)	λ	4-60*03	3*02	CETWDSNTRVF	4 (3)
34-1-4A	3-30*03 or 18 or 3-30-5*01	4*02	5-18*01	3	CTKDRVAYSYGPTRVDYW	12 (10)	κ	1-9*01	3*01	CQQLNSYPIFTF	4 (3)
34-3-2A	3-48*03	5*02	1-7*01	1	CARDGGVTGGTNPNWFDPW	2 (1)	λ	4-69*01	2*01 or 3*01	CQTWGTGIHVVF	1 (0)
34-3-6A	4-39*01	4*02	1-26*01	1	CARGIVGAYDYW	1 (1)	κ	1-5*03	1*01	CQQYNSYSWTF	1 (0)
34-3-8B	4-39*01	4*02	2-2*01	1	CASLRNQLLPHPHYW	5 (4)	λ	4-60*03	3*02	CETWDSNTQTF	1 (1)
34-1-8D	4-39*01	6*02	2-21*02	2	CARLYYGDYYSPRPSSPAYYYYGMDVW	5 (4)	λ	3-1*01	3*02	CQAWDSSTWVF	1 (0)
34-3-9B	7-4-1*02	4*02	2-15*01	2	CARGGHCSGRSCFYFDYW	20 (10)	λ	2-14*01	3*02	CTSYTTSRTWVF	19 (9)

The number of nucleotide mutations in the heavy and light chain variable domains and the number of amino acid replacements (shown in parentheses).

Abbreviations: V_H, variable gene segment of the heavy chain variable domain; D_H, diversity gene segment of the heavy chain variable domain; J_H, joining gene segment of the heavy chain variable domain; Mut, mutation number; $V_κ$, variable gene segment of the kappa light chain variable domain; $V_λ$, variable gene segment of the lambda light chain variable domain; $J_κ$, joining gene segment of the kappa light chain variable domain; $J_λ$, joining gene segment of the lambda light chain variable domain.

Supplementary Table 2. Binding activity of neutralizing EV71-specific human monoclonal antibodies.

mAb	12-96015 (B5)			11-96023 (C4)		
	EC_{99} ($\mu g\ ml^{-1}$)	EC_{50} ($\mu g\ ml^{-1}$)	Kd (nM)	EC_{99} ($\mu g\ ml^{-1}$)	EC_{50} ($\mu g\ ml^{-1}$)	Kd (nM)
16-3-10B	0.524	0.095	0.92 ± 0.12	0.346	0.064	0.37 ± 0.05
16-2-8C	0.829	0.165	1.76 ± 0.28	1.445	0.062	1.12 ± 0.16
16-2-11B	1.513	0.727	6.99 ± 1.52	0.638	0.318	2.85 ± 0.58
17-2-2B	0.359	0.061	1.12 ± 0.22	0.403	0.091	1.60 ± 0.33
16-2-9D	1.629	0.265	4.86 ± 0.85	2.963	0.083	1.10 ± 0.10
16-2-2D	0.733	0.111	1.73 ± 0.30	0.543	0.076	1.44 ± 0.26
17-1-12A	0.420	0.070	1.18 ± 0.22	0.203	0.029	0.49 ± 0.09
16-3-3C	0.540	0.080	1.40 ± 0.25	0.857	0.165	2.80 ± 0.53
16-2-12D	6.534	1.481	39.41 ± 9.33	1.135	0.199	3.65 ± 0.69
34-1-6D	1.363	0.161	2.66 ± 0.46	0.445	0.093	1.60 ± 0.32
16-3-4D	0.183	0.037	0.63 ± 0.13	0.228	0.050	0.83 ± 0.17
17-2-12A	3.572	0.640	12.81 ± 2.53	1.070	0.299	5.83 ± 1.27

Abbreviations: mAb, monoclonal antibody; EC, effective binding concentration.

Supplementary Table 3. Sequences of PCR primers.

Genotype B-VP4	Sense	ATCCGGTGTGCAATAGAGC
	Antisense	CATTCACCATAACCGACTATG
Genotype B-VP2	Sense	ACAGAGCCTCAAACAAGAC
	Antisense	GAAATTTGGCAGAATGGGTGC
Genotype B-VP3	Sense	ATCACTCTAGCTCCAATGTGCTC
	Antisense	CTACTCACACTGTCTCCTATAGAGC
Genotype B-VP1	Sense	ACCATGAAACTCTGCAAGG
	Antisense	GAAAAACTGACTGGGTAGTG
Genotype C-VP4	Sense	ATCCGGTGTGCAACAGAGC
	Antisense	CACTCACCATAACCGACTATG
Genotype C-VP2	Sense	ACAGAGTCTCAAGCAGGAT
	Antisense	GAAGTTTGGTAGAATAGGTGC
Genotype C-VP3	Sense	TACGACCAAGGAGCGACGC
	Antisense	CTGTGGGTGCTGGTAGAGC
Genotype C-VP1	Sense	GCGGCAGCCCAAAAGAA
	Antisense	AAGATTTGCCCAATCATTGTG

Supplementary Methods

Production of human monoclonal antibodies

Sorted single plasmablast cells were used to produce human monoclonal antibodies for further functional studies and repertoire analyses as previously described (12). Briefly, single plasmablast cells were sorted directly into RT-PCR catch buffer, and the variable domain genes from each cell were amplified in a one-step RT-PCR reaction (Qiagen) using a cocktail of sense primers specific for the leader region and antisense primers to the Cγ constant region for the heavy chain and Cκ and Cλ for the light chain. RT-PCR products were amplified in separate PCR reactions for the individual heavy and light chain gene families using nested primers to incorporate restriction sites at the ends of the variable gene as previously described (12). These variable genes were then cloned into expression vectors for the heavy and light chains. Plasmids were transfected into the 293T cell line (ATCC, CRL-3216) for the expression of recombinant human monoclonal antibodies in serum-free transfection medium. Full-length IgG1 human monoclonal antibodies produced in vitro were further analyzed regarding their antigen specificity and biochemical function.

Enzyme-linked immunosorbent assay

An ELISA plate (F96 Maxisorp NUNC-Immuno plate) was coated with purified viruses at the optimal concentration in carbonate buffer and incubated at 4°C overnight. The next day, unbound antigens were removed by pipetting to avoid the risk of forming aerosols. Nonspecific binding was blocked with a solution of PBS with 3% BSA at room temperature for 1 hour on a shaker. After removing the blocking buffer, the mAb-containing cell culture supernatant or purified mAb

preparation was added followed by incubation at 37°C for 1 hour. The non-transfected cell culture supernatant, the anti-influenza human monoclonal antibody 2-12C (in house), post-infection serum from a pediatric patient with laboratory-confirmed EV71 infection, and the anti-EV71 VP2 mouse monoclonal antibody MAB979 (EMD Millipore) were used as antibody controls for each experiment. After incubation, the plate was washed and incubated with HRP-conjugated rabbit anti-human or anti-mouse IgG (Rockland Immunochemicals) as a secondary antibody. After incubation, the plate was washed and developed with TMB substrate reagent (BD Biosciences). The reaction was stopped by adding 0.5 M HCl, and the optical density was measured at OD450 on a microplate reader.

Immunoprecipitation assay

For immunoprecipitation, Dynabeads® Protein G (Thermo Fisher Scientific) was prepared following the protocol and incubated with monoclonal antibody diluted in PBS/Tween-0.02% (5 μg ml^{-1}) with rotation for 10 minutes at room temperature. After removing unbound antibody and gently washing with PBS/Tween-0.02%, the beads-antibody complex was incubated with pre-cleared EV71-containing supernatants with rotation for 20 minutes at room temperature. After washing with PBS, the beads-antibody-antigen complex was re-suspended and transferred to a clean tube. The complex was incubated with elution buffer (50 mM, pH 2.8 glycine) at room temperature for 2 minutes. The eluted antibody and antigen were transferred to a clean tube, and the pH of the eluate was adjusted with 1 M Tris. The eluate was resolved by SDS-PAGE and analyzed by silver staining (Thermo Fisher Scientific) and western blotting. Here, for the western blot analysis, the eluate (antibody and antigen) was prepared under heated/reducing conditions, separated using SDS-PAGE,

and transferred to a nitrocellulose membrane. After blocking and washing, the membrane was incubated with the anti-VP2 mouse monoclonal antibody MAB979 (1:1000 dilution) (EMD Millipore) or anti-VP1 mouse monoclonal antibody MAB1255-M05 (1:1000 dilution) (Abnova). After washing, the membrane was incubated with HRP-conjugated goat anti-mouse IgG antibody (1:5000 dilution) (Thermo Fisher Scientific). After washing, the membrane was developed with peroxidase substrate for enhanced chemiluminescence (Thermo Fisher Scientific).

Western blotting

The EV71-containing supernatants were processed under heated/reducing and unheated/nonreducing conditions, separated using SDS-PAGE, and transferred to a nitrocellulose membrane by western transfer. Non-specific binding was blocked with 5% skimmed milk on a shaker at room temperature for 1 hour. The membrane was then probed with EV71-specific human mAbs (5 μg ml^{-1}) or the anti-VP2 mouse monoclonal antibody MAB979 (1:1000 dilution) (EMD Millipore) or anti-VP1 mouse monoclonal antibody MAB1255-M05 (1:1000 dilution) (Abnova) at 4°C overnight. After washing, the membrane was incubated with HRP-conjugated rabbit antibody to human IgG (1.25 μg ml^{-1}) (Dako) or HRP-conjugated goat anti-mouse IgG antibody (1:5000 dilution) (Thermo Fisher Scientific) at 4°C for 2 hours. After washing, the membrane was developed with peroxidase substrate for enhanced chemiluminescence (Thermo Fisher Scientific).

Selection of EV71 mutants with monoclonal antibodies

Wild-type plaque-purified 12-96015 and 11-96023 EV71 were diluted to a 50 $TCID_{50}$ x neutralization titer against 25 μg ml^{-1} mAb and incubated with an equal volume of mAb at a final concentration of 25 μg ml^{-1} for 1 hour at room temperature. The

mixture was then added to a flat-bottomed well containing a confluent monolayer of RD cells in viral growth medium (DMEM/2% FBS/penicillin and streptomycin) and incubated at 37°C for 4 days. If no cytopathic effect was observed, the cells and supernatant were collected, freeze-thawed three times, filtered, and then used to re-infect a freshly prepared RD cell layer at 37°C for 4 days. Once a cytopathic effect was observed in the first or second re-infection cycle, the cells and supernatant were collected and freeze-thawed three times. Cell debris was removed by centrifugation, the virus-containing solution was harvested, and the virus was then plaque purified. In the study, a cytopathic effect was usually observed in the first or second cycle of re-infection, and hence, the mAb-resistant mutant was developed. The virus was plaque purified, and the viral titer was determined by $TCID_{50}$ assay. Plaque-purified mAb-resistant mutants were confirmed by verifying the lack of mAb binding and neutralization activities.

Site-directed mutagenesis of the infectious clone

A site-directed mutagenesis study was carried out on an infectious cDNA clone of genotype B5 EV71 (strain N1745-TW08, GenBank accession number KT354870.1) (provided by Jen-Ren Wang, National Cheng Kung University, Taiwan) (2). A set of paired overlapping oligonucleotides was used to introduce nucleotide substitutions into the P1 gene of the infectious clone. The site-specific mutation in the infectious clone was generated using a QuikChange site-directed mutagenesis kit (Agilent). Sequencing of the P1 region of the mutant clone plasmid was performed, and the sequence was compared with that of the wild-type clone plasmid to confirm the introduction of the mutation. Plasmid DNAs of mutant clones were linearized by MluI (New England BioLabs), extracted with phenol/chloroform, precipitated with ethanol,

and then dissolved in RNase-free water. The size of the linear DNA was checked on an agarose gel. Then, 1 μg of the linear DNA template was used for in vitro transcription to generate viral genomic RNAs (Thermo Fisher Scientific). After precipitation and purification, 1 μg of the viral RNAs was transfected into 2 x 10^6 RD cells in 6-well plates using the Lipofectamine® 2000 reagent (Thermo Fisher Scientific). When cells showed 100% CPE, usually on day 3 to 4 post-transfection, the cells and supernatant were collected and freeze-thawed three times. Cell debris was removed by centrifugation, and the virus-containing solution was harvested as the first passage virus. Here, the P1 region of the mutated virus was sequenced, and the nucleotide and amino acid sequences were further compared with those of the wild-type virus to confirm the site-specific mutation. The viral titer was determined by $TCID_{50}$ assay.

Reagents and calculation of EC_{50} in the pre- and post-attachment neutralization assay

Antibodies and viruses were prepared in cold virus dilution medium (DMEM/10% FBS/penicillin and streptomycin). The wash procedure was carefully performed with cold virus dilution medium. For the 5-day incubation step, cells were incubated in virus growth medium (DMEM/2% FBS/penicillin and streptomycin).

At the end of the incubation, the cells were examined for the development of cytopathic effects. In addition, the viability of the cells was determined by the MTT (3-(4,5-dimethylthiazol-2-yl)-2,5-diphenyl tetrazolium bromide) assay. The optical density of the supernatant was measured at OD570 on a microplate reader. For each dilution of a given sample, percent neutralization was calculated as follows: [(value for antibody − value for virus only) / (value for cell only − value for virus only)]*100.

The 50% effective concentration was calculated by nonlinear regression analysis using GraphPad Prism software.

Sera study

The post-infection sera were collected from 27 pediatric patients with laboratory-confirmed EV71 infection in 2012 in Taiwan (13). The clinical EV71 isolates from these patients belonged to genotype B5 based on the VP1 analysis. Sera were stored at -80°C before testing.

Supplementary References

1. Luo, S.T. et al. Reemergence of enterovirus 71 epidemic in northern Taiwan, 2012. PLoS One 10, e0116322 (2015).

2. Huang, S. W. et al. Mapping Enterovirus A71 Antigenic Determinants from Viral Evolution. J. Virol. 89, 11500-11506 (2015).

3. Wang, X. et al. A sensor-adaptor mechanism for enterovirus uncoating from structures of EV71. Nat. Struct. Mol. Biol. 19, 424-429 (2012).

4. Plevka, P. et al. Structure of human enterovirus 71 in complex with a capsid-binding inhibitor. Proc. Natl. Acad. Sci. U S A 110, 5463-5467 (2013).

5. Tan, C. W., Sam, I. C., Lee, V. S., Wong, H. V. & Chan, Y. F. VP1 residues around the five-fold axis of enterovirus A71 mediate heparan sulfate interaction. Virology 501, 79-87 (2016).

6. Nishimura, Y. et al. Enterovirus 71 binding to PSGL-1 on leukocytes: VP1-145 acts as a molecular switch to control receptor interaction. PLoS Pathog. 9, e1003511 (2013).

7. Chen, P. et al. Molecular determinants of enterovirus 71 viral entry: cleft around GLN-172 on VP1 protein interacts with variable region on scavenge receptor B 2. J. Biol. Chem. 287, 6406-6420 (2012).

8. Ku, Z. et al. Single Neutralizing Monoclonal Antibodies Targeting the VP1 GH Loop of Enterovirus 71 Inhibit both Virus Attachment and Internalization during Viral Entry. J. Virol. 89, 12084-12095 (2015).

9. Dang, M. et al. Molecular mechanism of SCARB2-mediated attachment and uncoating of EV71. Protein Cell 5, 692-703 (2014).

10. Li, X. et al. Generation of neutralizing monoclonal antibodies against Enterovirus 71 using synthetic peptides. Biochem. Biophys. Res. Commun. 390, 1126-1128 (2009).

11. Lim, X. F. et al. Characterization of an isotype-dependent monoclonal antibody against linear neutralizing epitope effective for prophylaxis of enterovirus 71 infection. PLoS One 7, e29751 (2012).

12. Huang, K. Y. et al. Focused antibody response to influenza linked to antigenic drift. J. Clin. Invest. 125, 2631-2645 (2015).

13. Huang, K. Y. et al. A Potent Virus-Specific Antibody-Secreting Cell Response to Acute Enterovirus 71 Infection in Children. J. Infect. Dis. 212, 808-817 (2015).
